# Trust in medical art is the most effective coping mechanism for predicting treatment satisfaction in elective neurosurgery

**DOI:** 10.1038/s41598-026-43341-x

**Published:** 2026-03-10

**Authors:** Lisa Schock, Lilith Philomena Laflör, Džiugas Meška, Adrian Engel, Hanah Hadice Karadachi, Emad Mohajerani, Laurèl Rauschenbach, Marvin Darkwah Oppong, Ramazan Jabbarli, Philipp Dammann, Sonja Siegel, Ilonka Kreitschmann-Andermahr, Ulrich Sure, Yahya Ahmadipour

**Affiliations:** 1 Department of Neurosurgery and Spine Surgery, University Hospital Essen, University of Duisburg-Essen, Hufelandstraße 55, 45147 Essen, Germany; 2 Center for Translational Neuro- & Behavioral Sciences (C-TNBS), University of Duisburg-Essen, Essen, Germany; 3 German Cancer Consortium (DKTK) Partner Site, University Hospital Essen, University of Duisburg-Essen, Essen, Germany

**Keywords:** Patient expectations, Neurosurgery, Coping strategies, Patient education, Treatment satisfaction, Diseases, Health care, Medical research, Neurology, Neuroscience

## Abstract

Patient expectations and satisfaction are critical outcomes in cranial and spinal neurosurgery, yet discrepancies between anticipated and actual results can hinder postoperative adjustment. This prospective longitudinal study investigated how coping strategies and preoperative patient education influence satisfaction with surgical outcomes. Two self-developed questionnaires assessed expectations, perceived illness burden, patient education quality, hospital experience, postoperative recovery, and overall satisfaction after surgery. Coping strategies were evaluated using the validated *Essen Coping Questionnaire*. Data from 277 patients were analyzed, stratified by surgical complexity. No significant difference in patient education quality was found between complexity groups (mean difference 0.62, 95% CI [–1.65, 0.41]; *t*(221) = –1.19, *p* = .237). Patients undergoing less complex procedures reported higher preoperative burden (mean difference 1.72, 95% CI [–3.33, –0.10]; *t*(231) = –2.09, *p* = .038). Linear regression revealed that the quality of preoperative education, postoperative burden, and coping strategies *trust in medical art* and *willingness to accept help* significantly predicted satisfaction (*F*(10,79) = 3.41, *p* < .001). These findings highlight the importance of patient-doctor communication, tailored education, and psychological preparedness in shaping patient-reported outcomes. Enhancing preoperative support and fostering adaptive coping may improve satisfaction and postoperative adjustment, advocating for a more personalized approach to neurosurgical care.

**Supplementary Information:**

The online version contains supplementary material available at 10.1038/s41598-026-43341-x.

## Introduction

Patient expectations and satisfaction are now widely recognized as essential components in evaluating outcomes in spinal surgery^1–3^. While traditional outcome measures have focused on objective clinical metrics, modern research emphasizes understanding and addressing patients’ preoperative expectations. These expectations shape postoperative experience, satisfaction, and perceived quality of care^2,4^. Unfortunately, the measurement of those parameters rarely exists in elective cranial neurosurgery^5,6^.

Before surgery, patients commonly hold high expectations for postoperative improvement, particularly in terms of pain relief, functional recovery, and quality of life^1,7^. For instance, in functional neurosurgery, patients may have hopes that far exceed what is realistic, which can create a gap between desired and actual outcomes and complicate postoperative adjustment^1^. Similarly, while most spinal surgery patients experience clinically relevant improvements, only approximately half of these patients report that their most important expectations are fully met^4,8^. Despite this discrepancy, satisfaction levels often remain high, suggesting that factors beyond expectation fulfillment—such as the importance patients attach to recovery and the degree of improvement they experience—contribute to shaping their perceptions^7,8^.

The relationship between expectations, outcomes, and satisfaction is complex and not fully understood. Some studies find that higher preoperative expectations are associated with greater satisfaction. Other studies report that satisfaction is more closely linked to actual functional improvement than to the fulfillment of specific expectations^4,8^. Furthermore, it was found that unmet expectations can lead to dissatisfaction, which highlights the need for better preoperative communication and expectation management^2,4^. Validated questionnaires are increasingly used as tools to systematically assess patient expectations, guide clinical discussions, and tailor postoperative support^1,3^.

Coping strategies have been shown to essentially influence the psychological well-being when experiencing a chronic disease^9–11^. In the neurosurgical setting, the effectiveness of coping strategies to deal with surgery-related anxiety was examined before surgery^12^: The coping strategy *Optimism and Trust* was the most frequently reported dimension and it was positively related to psychosocial well-being. Further research is required to understand the patients’ burden and needs in the neurosurgical setting.

The present work sets the stage for a deeper exploration of how patient expectations are formed, measured, and managed in neurosurgical and spinal surgery settings and how these factors influence patient-reported outcomes and satisfaction. Specifically, we investigated how coping strategies and patient education before surgery influence the satisfaction with the surgery outcome.

## Subjects, materials and methods

### Subjects

The present work is a prospective, longitudinal monocentric study. Patients were tested before and after neurosurgery or spine surgery. Data was collected between February 2024 and August 2025. Clinical and radiological data of patients were collected from the medical records. Patients who were unable to complete the questionnaires or assess their current situation adequately due to a language barrier, or due to severe physical or neuropsychological impairments, were excluded. The study included all patients admitted to the neurosurgery unit for elective procedures. Neuroradiological interventions were excluded from the analysis, as they differ fundamentally from neurosurgical procedures in terms of technique, invasiveness, and clinical indication.

The study was approved by the local ethics committee (23-11643-BO) and was conducted according to the tenets of the Declaration of Helsinki. All patients gave their written informed consent before participation in the study.

### Testing procedure

On the day of admission, preoperative questionnaires were handed out to the participants. It took about 20–30 min to complete the forms.

The postoperative questionnaires were mailed to the participants’ homes and were received with a median (IQR) of 4 weeks (3.00–6.00) after preoperative data collection, which represents the transition phase from acute recovery to early rehabilitation.

### Questionnaires

A self-made preoperative questionnaire assessed the burden and expectation regarding seven categories (Strength, Movement, Senses, Mental state, Emotional state, Communication, Family life) as well as five questions on the doctor-patient informative talk on the surgery. The questionnaire was designed to reflect domains that patients and clinicians identified as clinically relevant. These domains were selected based on qualitative feedback from patients and multidisciplinary team discussions, ensuring clinical relevance and patient-centeredness.

The burden was rated on a five-point Likert scale with emotional smiley faces (coded 0–4), ranging from “very strongly burdened” (0, very negative smiley) to “not at all burdened” (4, very positive smiley). The expectation on surgery outcome was measured using a five-point Likert scale indicating the anticipated duration of impairments in the same seven domains, with the response options *not applicable, days, weeks, months, and years*.

The postoperative questionnaire contained the same items as for the burden and expectation part. Instead of the five questions about the doctor-patient informative talk, there were ten items on the satisfaction with the hospital stay and the surgery outcome. The complete questionnaires including all items and response formats are provided in the Supplementary Material S1.

From these questionnaires, six composite scores were calculated: a burden score preoperative and postoperative (sum scores, maximum value 28 each), an expectation score preoperative and postoperative (mean scores across seven items), an education score preoperative (sum score, maximum value 20), and a satisfaction score postoperative (sum score, maximum value 40). For the expectation scores, the values were averaged across the seven domains to reflect the mean expected duration of impairments after surgery, coded from *not applicable* to *years*. Lower values correspond to shorter expected impairment durations, while higher values indicate expectations of longer-lasting impairments. All other scores were computed as simple sum scores. The postoperative expectation score additionally served as a control variable to test whether the surgery changed patients’ recovery expectations. In all scales, higher scores represent more favorable outcomes, namely less burden, more satisfaction, and better patient education.

The Essen Coping Questionnaire (Essener Fragebogen zur Krankheitsverarbeitung, EFK) is a brief cross-disease screening measure, originally developed for visually impaired patients. It consists of 45 items that are rated on a five-point Likert scale ranging from 0 (“not at all”) to 4 (“very strongly”), thereby capturing how strongly a coping strategy applies to the respondent. Using these items, the EFK measures the following nine coping domains at the emotional, cognitive, and behavioral levels: (1) Action-oriented, problem-focused coping, (2) Distance and self-construction, (3) Information-seeking and experience-sharing, (4) Trivialization, wishful thinking, and threat defense, (5) Depressive processing, (6) Willingness to accept help, (7) Active search for social inclusion, (8) Trust in medical art, (9) Working out an inner support. Each domain is represented by several items, and the corresponding subscale scores are computed as mean values of the item responses, with higher values indicating a stronger use of the respective coping strategy. Normative data are available from N = 1,815 chronically ill patients^13^.

### Data analysis

Data analysis was performed using IBM SPSS Statistics 30.0.0.0.

Descriptive statistics were performed for sample characteristics and questionnaire scores.

For the attrition analyses, group comparisons between completers and dropouts were conducted using Fisher´s exact test or independent samples *t*-test.

To balance group sizes and account for procedural complexity, surgical cases were divided into two groups by a neurosurgery specialist based on operative duration. Procedures lasting two hours or less were classified as lower complexity, while those exceeding two hours were classified as higher complexity.

A normal distribution of the data was assumed due to the large sample size. Missing values were regarded as missing at random and were deleted case-wise from the analysis. Datapoints outside of the triple interquartile range were defined as extreme outliers. Group comparisons were performed using two-sample *t*-test (two-tailed). Correlations were conducted with Pearson’s correlation coefficient *r*.

A multiple stepwise linear regression model with the predictors coping strategies and type of intervention, and satisfaction with the outcome of the surgery as dependent variable were applied.

In a second regression analysis, the predictors education, burden and expected recovery time before surgery, as well as burden and expected recovery time after surgery were tested with the outcome variable satisfaction with the outcome of the surgery. Assumptions of the linear regression model were fulfilled. Effect sizes (*r*) were categorized according to Cohen^14^. Statistical significance was set at an α-level of *p* < .050.

## Results

### Sample and surgery categories

**Fig. 1:**
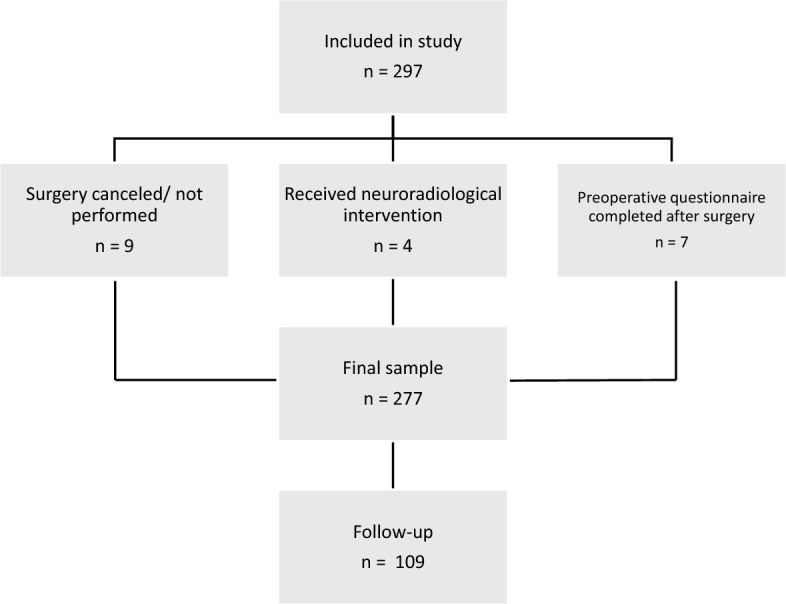
Flow of study participants. Of the 297 patients initially included in the study, 9 did not undergo surgery, and 4 received neuroradiological rather than neurosurgical treatment. Additionally, 7 participants completed the preoperative questionnaire only after surgery and were excluded due to timing mismatch with the intended measurement point. The final sample consisted of 277 patients with preoperative data, 109 of whom were in the follow-up.

A total of 277 patients were included. Figure 1 illustrates the exclusion process and final sample.

In Table 1, sample characteristics are listed.

**Table 1:** Patient characteristics for the whole group and divided by completers and dropouts.

Variable	*M (SD)*	Frequency	Completers	Dropouts	*p*-value*
Sex m/f		116/161	43/66	73/95	.535
BMI	28.40 (5.61)		28.97 (6.16)	28.00 (5.17)	.180
Age	55.27 (14.47)		56.50 (13.24)	54.48 (15.19)	.245
Relapse y/n		58/209	30/77	28/132	.049*
Years of education	14.70 (2.97)		14.74 (3.36)	14.67 (2.52)	.893
Tumor y/n		175/102	71/38	104/64	.612

The table presents demographic and clinical characteristics for the whole group and divided by completers and dropouts. *p* < .050. *m/f* = male/female; *y/n* = yes/no; *M* = mean, *SD* = standard deviation, * statistical significance of the comparison of completers and dropouts.

Surgery types can be found in Table 2. A high rate of microsurgical tumor resection is noteworthy.

**Table 2:** Surgical interventions.

Surgical complexity	Type of surgical intervention	Frequency	Percentage	Total percentage
Lower complexity	Ventriculoperitoneal shunt implantation	2	0.7%	48.4%
	Peripheral nerve surgery	4	1.4%	
	Soft tissue surgery	2	0.7%	
	Implantation of a drug pump	1	0.4%	
	Cranioplasty	8	2.9%	
	Endoscopic extirpation	23	8.3%	
	Stereotactic biopsy	17	6.1%	
	Cervical corporectomy	2	0.7%	
	Foraminotomy	5	1.8%	
	Laminoplasty	4	1.4%	
	Hemilaminectomy	17	6.1%	
	Cervical ventral fusion	14	5.1%	
	Laminectomy	7	2.5%	
	Discectomy	7	2.5%	
	Metal removal	2	0.7%	
	Microsurgical decompression	5	1.8%	
	Interlaminar fenestration	14	5.1%	
Higher complexity	Microsurgical tumor resection	128	46.2%	51.6%
	Aneurysm clipping	1	0.4%	
	Deep brain stimulation	1	0.4%	
	Spondylodesis	10	3.6%	
	Transforaminal lumbar interbody fusion (TLIF)	3	1.1%	

Sample characteristics in terms of the surgical intervention, divided by complexity (lower, higher).

### Pre- and postoperative scores of self-constructed questionnaires

Patients scheduled for less complex surgery experienced a significantly higher burden before surgery than those scheduled for higher complexity in procedures with a mean burden score difference of 1.72 (95%-CI[− 3.33, − 0.10]; *t*(231) = − 2.09, *p* = .038). However, the burden after surgery did not differ between these groups with a mean score difference of 0.76 (95%-CI[-2.93,1.42]; *t*(99) = − 0.69, *p* = .491).

**Fig. 2:**
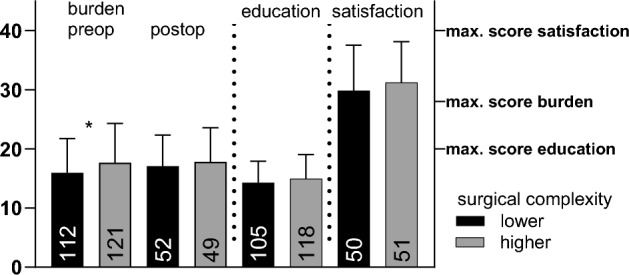
Burden (pre- vs. postoperative), education (preoperative) and satisfaction (postoperative) scores. Mean of sum scores before and after surgery (pre- and postoperative), divided by groups of lower and higher complexity. Error bars represent standard deviation (SD). Higher values represent less burden. *p < .05. *n* is shown as annotation.

The quality of patient education before surgery did not differ between the two groups receiving different types of procedures with an education score difference of 0.62 (95%-CI[− 1.65, 0.41]; *t*(221) = − 1.19, *p* = .237); the same applies to the satisfaction score postoperative with a mean satisfaction score difference of 1.38 (95%-CI[− 4.26,1.51]; *t*(99) = − 0.95, *p* = .347. Compare Fig. 2.

**Fig. 3:**
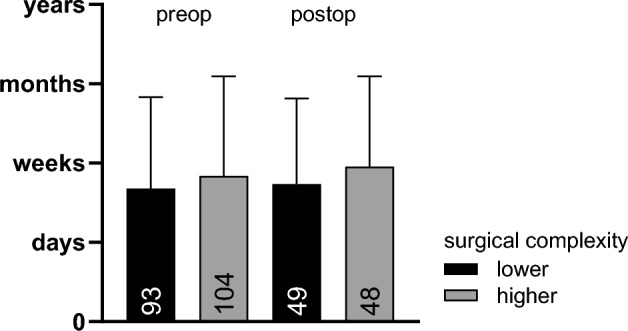
Expectation score (pre- vs. postoperative). Mean of patients’ expectation of the anticipated duration of impairments before and after surgery (pre-post expectation), divided by groups of lower and higher complexity. Error bars represent standard deviation (*SD*). *n* is shown as annotation.

The expectation of the time until recovery before surgery did not differ between the two groups receiving different types of procedures with an expectation score difference of 0.16 (95%-CI[− 0.50, 0.18]; *t*(195) = − 0.93, *p* = .354); the same applies to the expectation score postoperative with a mean score difference of 0.22 (95%-CI[− 0.67,0.23]; *t*(95) =  − 0.98, *p* = .330. Compare Fig. 3.

When comparing completers and dropouts, burden scores with a mean difference of − 0.62 (95%-CI[− 2.26,1.02]; *t*(231) = − 0.74, *p* = .458), patient education scores (mean difference − 0.24; 95%-CI[− 1.27,0.80]; *t*(221) =  − 0.45, *p* = .654) and expectation scores (mean difference 0.11; 95%-CI[− 0.24,0.45]; *t*(195) = 0.61, *p* = .540) did not differ before surgery.

### Descriptives of coping strategies

Compared to the normative values of chronically ill patients, the present cohort showed similar results, except for the *trust in medical art* scale, which showed a mean higher than average. Confer to Table 3.

**Table 3:** Descriptive values of coping strategies from the EFK for the whole group, with reference values from a German normative population.

Scale	*M*	*SD*	Ref. *M*	Ref. *SD*
Action-oriented, problem-focused coping	2.46	.81	2.24	.88
Distance and self-construction	2.10	.70	1.96	.78
Information-seeking and experience-sharing	1.19	.85	1.42	.93
Trivialization, wishful thinking, and threat defense	1.08	.66	1.22	.71
Depressive processing	0.87	.71	0.71	.70
Willingness to accept help	1.62	.80	1.48	.76
Active search for social inclusion	1.83	.81	1.93	.89
Trust in medical art	3.30	.58	2.46	.79
Working out an inner support	1.14	.71	1.32	.81

*M* = mean; *SD* = standard deviation; *Ref* = reference; Minimum score 0, maximum score 4.

The comparison of completers and dropouts yielded no significant difference in any of the coping strategies with mean differences ranging from 0.01 for the scale *Trivialization, wishful thinking, and threat defense* (95%-CI[− 0.17,0.18]; *t*(224) = − 0.09, *p* = .927) to − 0.09 for the scale *working out an inner support* (95%-CI[− 0.28,0.09]; *t*(222) = − 0.98, *p* = .326).

### Correlation of coping strategies with quality of patient education before surgery

Positive correlations with the quality of the preoperative education by the neurosurgeon were obtained for the coping strategies *action-oriented problem-focused coping* (*r* = .210, *p* = .003), *active search for social inclusion* (*r* = .251, *p* =  < .001), and *trust in medical art* (*r* = .422, *p* =  < .001). A negative correlation was found for the coping strategy *depressive processing* (*r* = − .182, *p* = .008). Effect sizes are to be classified as small, with the only moderate one being *trust in medical art*.

### Prediction of satisfaction with treatment

In the first step of the regression model, which included only surgery category (OP category 1/2 vs. 3), the regression was not significant, *F*(1, 88) = 0.80, *p* = .375, with an *R*^2^ of .009.

When coping strategies were added in the second step, the regression was significant, *F(*10, 79) = 3.41, *p* < .001, with an *R*^2^ of .302 (adjusted *R*^2^ = .213). This indicates that coping strategies explained an additional 29% of the variance in postoperative satisfaction scores beyond surgery category.

Within this model, *willingness to accept help* (*B* = − 2.89, *p* = .016) and *trust in medical art* (*B* = 5.90, *p* < .001) emerged as significant predictors. None of the other coping scales were significant. Table 4 presents the regression coefficients for both steps of the model.

**Table 4:** Stepwise regression model for the prediction of the postoperative satisfaction score.

Predictor	*B*	*SE B*	*β*	*t*	*p*	95% CI LL	95% CI UL
Step 1							
Constant	29.84	1.11	–	26.88	< .001	27.64	32.05
Surgery category	1.38	1.55	.10	0.89	.375	− 1.70	4.46
Step 2							
Constant	14.33	4.90	–	2.92	.005	4.57	24.08
Surgery category	1.18	1.41	.08	0.84	.403	− 1.61	3.98
Coping strategy							
Action-oriented	− 0.51	1.06	− .06	− 0.49	.629	− 2.62	1.59
Distance	0.39	1.32	.04	0.29	.772	− 2.25	3.02
Information-seeking	1.70	0.97	.20	1.76	.083	− 0.23	3.62
Trivialization	− 1.21	1.13	− .11	− 1.07	.288	− 3.46	1.04
Depressive	− 0.69	1.17	− .07	− 0.59	.559	− 3.01	1.64
Accept help	− 2.89	1.17	− .32	− 2.46	.016	− 5.22	− 0.56
Social inclusion	0.57	1.12	.06	0.51	.609	− 1.65	2.80
Trust in medical art	5.90	1.37	.47	4.31	< .001	3.17	8.63
Inner support	0.11	1.16	.01	0.10	.923	− 2.20	2.43

*B* = unstandardized regression coefficient; *SE B* = standard error; *β* = standardized regression coefficient; CI = confidence interval; LL = lower limit; UL = upper limit.

In the second regression model predicting postoperative satisfaction, five predictors were entered simultaneously: preoperative burden, postoperative burden, preoperative expectations, postoperative expectations, and the quality of the preoperative consultation with the neurosurgeon.

The model was significant,* F*(5, 81) = 5.74, *p* < .001, with an *R*^2^ of .261 (adjusted *R*^2^ = .216), indicating that approximately 26% of the variance in postoperative satisfaction could be explained by the set of predictors.

Within the model, postoperative burden (*B* = 0.63, *p* < .001) and the preoperative consultation score (*B* = 0.49, *p* = .014) were significant predictors of satisfaction. Specifically, greater burden after surgery was associated with lower satisfaction, while more positive ratings of the preoperative consultation predicted higher satisfaction.

None of the other variables—preoperative burden, preoperative expectations, or postoperative expectations—were significant predictors in this model. Table 5 presents the regression coefficients and confidence intervals for all predictors in the model.

**Table 5:** Regression model for the prediction of the postoperative satisfaction score.

Predictor	*B*	*SE B*	*β*	*t*	*p*	95% CI LL	95% CI UL
Constant	15.50	3.64	–	4.26	< .001	8.27	22.74
Preoperative							
Education	0.49	0.20	.26	2.52	.014	0.10	0.88
Burden	− 0.19	0.13	− .16	− 1.40	.165	− 0.45	0.08
Expectations	0.64	0.65	.11	0.98	.329	− 0.65	1.93
Postoperative							
Burden	0.63	0.15	.47	4.20	< .001	0.33	0.93
Expectations	− 0.57	0.72	− .09	− 0.80	.427	− 2.00	0.85

*B* = unstandardized regression coefficient; *SE B* = standard error; *β* = standardized regression coefficient; CI = confidence interval; LL = lower limit; UL = upper limit.

## Discussion

In the present study, questionnaires on expectation, burden, preoperative patient education and coping strategies as well as satisfaction with the surgical outcome were administered to a large cohort of patients in elective neurosurgery. Patients undergoing less complex surgical procedures experienced a higher burden prior to the intervention than those undergoing complex surgeries. However, this discrepancy was no longer evident postoperatively. The quality of preoperative patient education was found to be consistent across both groups. Similarly, there was no discrepancy in the anticipated duration of symptom recovery between the two groups. The following factors were found to be significant predictors of postoperative satisfaction: the coping strategies *trust in medical art,* and *willingness to accept help*, the preoperative talk with the neurosurgeon and the postoperative burden were significant predictors of postoperative satisfaction.

Previous studies reported that younger patients (18–39 years old) with a primary central nervous system tumor often report higher symptom burden than older patients (≥ 40 years old)^15^ and that the psycho-oncological burden is high in patients with brain tumors and brain metastases^16,17^. Patients undergoing spinal neurosurgery typically experience severe pain, disability, fatigue, and psychological distress prior to surgery; a longer duration of symptoms and untreated depression or anxiety further increase the burden and predict poorer clinical outcomes^18^.

We did not focus on distinct aspects of burden in the self-developed questionnaire, but included questions on all aspects of symptoms (e.g. movement, cognition), to estimate a general level of burden. Spinal patients, who are over represented in the less complex intervention group, may experience a higher rate of pain and, consequently a higher burden^19^.

The measurement of pain should be included in further trials to account for the specific effects in the burden of different types of surgeries.

There was no difference in the quality of patient education before surgery between the two groups. This implies that patients felt similarly well-informed about the surgical procedure, its duration, and the risks and potential impairments after surgery, regardless of the complexity of the surgery. Furthermore, the quality of preoperative patient education predicted postoperative satisfaction.

Effective doctor-patient communication strongly moderates patient satisfaction. In line with results of the present study, previous research showed that patients who feel comfortable with and well-informed by their neurosurgeon are more likely to be satisfied with their treatment and less anxious before surgery, with anxiety being an important mediator of satisfaction^20,21^. In particular, Harrison et al. showed that patients who were satisfied with their experience felt that they were thoroughly informed, that they had a comprehensive understanding of the extent of the intervention and they knew what to expect during the early postoperative recovery period including timely information about hospital discharge^22^. Similarly, the present study included questions about discharge and length of stay as part of the postoperative satisfaction score.

There is evidence that postoperative satisfaction in lumbar spinal fusion surgery is shaped by actual surgery outcome, not the expectation of the patient^23^, but the majority of studies speak in favor of an influence of patient expectations in spine surgery^24^. Efforts are also made to enhance patient satisfaction and facilitate patient education in cranial surgery^25^.

Managing expectations is critical. Unrealistic or poorly managed expectations can lead to dissatisfaction, even if the surgical outcome is favorable^26^.

Shared decision-making, which involves patients in choosing their treatment options, can improve satisfaction; however, the direct impact on outcomes is still being studied^27^.

Additionally, the use of advanced tools, such as 3D virtual reality, during consultations are shown to be effective enhancers of patient knowledge^20^ and should be further investigated in the context of patient satisfaction and coping strategies.

The coping strategies in the present cohort corresponded to the mean scores of the normative population, except for the scale *trust in medical art*, which lay outside one standard deviation of the mean. That suggests that, in the present cohort, patients have high trust in the skills of the medical staff. There was a moderate correlation of the coping strategy t*rust in medical art* and the quality of the preoperative patient education with the neurosurgeon. There may be a reciprocal influence of pre-existing coping mechanism and the quality of the patient education, probably boosting the trust further^12^. Conversation with medical staff to cope with surgery related anxiety was shown before^28^. Patients surveyed on the day of admission are often in a state of heightened anxiety and dependency, which may lead to an overestimation of trust as a coping mechanism. This ‘preoperative trust bias’ could be a form of cognitive dissonance reduction, where patients affirm trust in the surgeon to manage uncertainty and fear. While this may reflect genuine confidence, it may also be transient and influenced by the immediate clinical context.

Coping strategies as predictors of burden and satisfaction in neurosurgery are strongly underrepresented in the current literature. One study reported that the mode of coping was related to fatigue in patients with aneurysmal subarachnoid hemorrhage^9^. In pituitary tumors, there was found avoidant coping^29^ and an influence of maladaptive coping styles on quality of life in acromegaly^11^.

In the present cohort, the strong prediction of satisfaction after surgery by coping strategies that mediate trust and acceptance of help implies that building this trust beforehand is crucial by conveying necessary information to patients in a tailored manner. To find out, how this interaction is shaped, remains a challenge for future research.

To our best knowledge, our study is the first in the field to deliver a combined examination of preoperative patient education and coping strategies with postoperative treatment satisfaction in a large cohort of neurosurgical patients.

Limitations of the study are the lack of assessment of anxiety, which would of course be an important influence factor on all outcome variables. Furthermore, the explicit assessment of pain would be an interesting factor concerning treatment expectations^30^ and coping strategies. The employment of a self-developed questionnaire limits direct comparability with other studies. In the future, researchers should validate these scales using established instruments. Despite the patients having been informed about data privacy, it cannot be ruled out that their response behavior was influenced by social desirability. Although attrition was substantial (60%), we found no significant differences between completers and dropouts in baseline demographic or psychological variables, suggesting that the results are unlikely to be biased by non-response. Patients with recurrent disease were more likely to complete the follow-up investigation, potentially reflecting more awareness to the importance of research in the field of their condition. The 4-week follow-up may not reflect long-term satisfaction. Future studies should include longer-term assessments (e.g., 3–6 months) to evaluate the stability of these findings. Furthermore, future studies should include an analysis of the neurosurgeon’s approach to educating patients to understand factors such as time spent with patients, choice of words, and setting. The use of operative complexity for group classification may have conflated procedural invasiveness with underlying pathology. However, because of the diverse range of brain and spine conditions, including both neoplastic and non-neoplastic disorders, stratification by pathology was not feasible. This approach would have resulted in small, imbalanced subgroups and limited statistical power.

Despite the complexities of patient expectations and satisfaction in neurosurgical and spinal surgery settings, our study emphasizes the significance of effective communication and coping strategies in improving patient-reported outcomes. Tailoring preoperative discussions and postoperative support to individual patient needs can help healthcare providers improve satisfaction and outcomes. Further research is needed to fully understand the relationship between expectations, coping strategies, and satisfaction, but our findings suggest that a more personalized approach to care can lead to better results.

## Supplementary Information

Supplementary Information.

## Data Availability

The datasets generated during and/or analyzed during the current study are available from the corresponding author on reasonable request.
