# Supplementary material for: Trust in medical art is the most effective coping mechanism for predicting treatment satisfaction in elective neurosurgery
Source: Sci Rep. 2026 Mar 10;16:8733. doi: 10.1038/s41598-026-43341-x (PMC12979813; doi:10.1038/s41598-026-43341-x)
Supplement: Supplementary file 1 — Supplementary Information. [file 41598_2026_43341_MOESM1_ESM.pdf]

Preoperative Expectations Questionnaire

In this questionnaire, we would like to find out about your current state of health and any associated stress, as well as your expectations regarding the treatment outcome, and compare this with the actual treatment outcome. The information obtained will help us to improve the treatment we offer. We also ask about your satisfaction with the information you received about the treatment.

It is important that you answer the questions in relation to your current condition. If, for example, you have a pre-existing mobility impairment (e.g., due to hip/knee problems), you should not take this into account, as we do not treat these conditions.

The **expected duration** after surgery refers to the period of time during which you will still have limitations after the surgery.

Please answer all questions. Please do not give your name.

Gender____________Age________________Height ______________Weight______________________

Sick leave since _____________Years of education (school+professional training)_________________

Recurrence (reappearance of the disease/relapse): yes/no; how many times____________________

Additional health issues ____

____

Strength

How burdened/restricted do you feel?	☐	☐	☐	☐	☐
	N.a. *	**Days**	**Weeks**	**Months**	**Years**
Expected persistence after surgery?	☐	☐	☐	☐	☐

*not applicable

Movement control Walking and running, (fine) motor skills of the hands, facial motor skills

How burdened/restricted do you feel?	☐	☐	☐	☐	☐
	N.a.	**Days**	**Weeks**	**Months**	**Years**
Expected persistence after surgery?	☐	☐	☐	☐	☐

Senses sight, hearing, touch, taste, smell, balance

How burdened/restricted do you feel?	☐	☐	☐	☐	☐

	N.a.	**Days**	**Weeks**	**Months**	**Years**
Expected persistence after surgery?	☐	☐	☐	☐	☐

Mental state Mood, balance

How stressed/restricted do you feel?	☐	☐	☐	☐	☐

	N.a.	**Days**	**Weeks**	**Months**	**Years**
Expected persistence after surgery?	☐	☐	☐	☐	☐

Cognitive performance Memory, attention, concentration

How stressed/restricted do you feel?	☐	☐	☐	☐	☐

	N.a.	**Days**	**Weeks**	**Months**	**Years**
Expected persistence after surgery?	☐	☐	☐	☐	☐

Communication skills Speech production, speech comprehension, reading, and writing

How burdened/restricted do you feel?	☐	☐	☐	☐	☐

	N.a.	**Days**	**Weeks**	**Months**	**Years**
Expected persistence after surgery?	☐	☐	☐	☐	☐

Family life

How burdened/restricted do you feel?	☐	☐	☐	☐	☐

	N.a.	**Days**	**Weeks**	**Months**	**Years**
Expected persistence after surgery?	☐	☐	☐	☐	☐

Information about the disease and treatment

Please complete this section after you have been informed by your doctor!

**How would you rate your knowledge of potential current physical limitations?**					
Current level of knowledge	☐	☐	☐	☐	☐
**How would you rate your knowledge of potential current mental limitations?**					
Current level of knowledge	☐	☐	☐	☐	☐
**How would you rate your knowledge of potential risks and complications?**					
Current level of knowledge	☐	☐	☐	☐	☐
**How would you rate your knowledge of the recurrence of the disease?**					
Current level of knowledge	☐	☐	☐	☐	☐
**How would you rate your knowledge of the surgical procedure and its duration?**					
Current level of knowledge	☐	☐	☐	☐	☐

Thank you for your cooperation. ☺

Postoperative expectations questionnaire

In this questionnaire, we would like to find out about your current state of health and the associated strain on you, and compare this with your expectations regarding the treatment outcome. We also ask about your satisfaction with your stay in hospital. The information obtained will be used to help improve the treatment we offer.

It is important that the assessment of your current condition only relates to your current illness. If, for example, you have a pre-existing mobility impairment (e.g., due to hip/knee problems), you should not take this into account, as we have not treated this.

Please complete the questionnaire <u>no later than two months</u> after the end of your hospital stay.

Please answer <u>all</u> questions. Please do not give your name.

Gender____________Age________________Height ______________Weight______________________

Sick leave since _____________Years of education (school+professional training)_________________

Recurrence (reappearance of the disease/relapse): yes/no; how many times____________________

Additional health issues ____

____

<u>Strength</u>

How burdened/restricted do you feel?	☐	☐	☐	☐	☐
	N.a. *	**Days**	**Weeks**	**Months**	**Years**
Expected persistence after surgery?	☐	☐	☐	☐	☐

*not applicable

<u>Movement control</u> Walking and running, (fine) motor skills of the hands, facial motor skills

How burdened/restricted do you feel?	☐	☐	☐	☐	☐
	N.a.	**Days**	**Weeks**	**Months**	**Years**
Expected persistence after surgery?	☐	☐	☐	☐	☐

<u>Senses</u> sight, hearing, touch, taste, smell, balance

How burdened/restricted do you feel?	☐	☐	☐	☐	☐
	N.a.	**Days**	**Weeks**	**Months**	**Years**
Expected persistence after surgery?	☐	☐	☐	☐	☐

<u>Mental state</u> Mood, balance

How stressed/restricted do you feel?	☐	☐	☐	☐	☐
	N.a.	**Days**	**Weeks**	**Months**	**Years**
Expected persistence after surgery?	☐	☐	☐	☐	☐

<u>Cognitive performance</u> Memory, attention, concentration

How stressed/restricted do you feel?	☐	☐	☐	☐	☐
	N.a.	**Days**	**Weeks**	**Months**	**Years**
Expected persistence after surgery?	☐	☐	☐	☐	☐

<u>Communication skills</u> Speech production, speech comprehension, reading, and writing

How burdened/restricted do you feel?	☐	☐	☐	☐	☐
	N.a.	**Days**	**Weeks**	**Months**	**Years**
Expected persistence after surgery?	☐	☐	☐	☐	☐

<u>Family life</u>

How burdened/restricted do you feel?	☐	☐	☐	☐	☐
	N.a.	**Days**	**Weeks**	**Months**	**Years**
Expected persistence after surgery?	☐	☐	☐	☐	☐

Hospital stay

How satisfied were you with the communication from the doctors...?

...in relation to your own concerns and wishes?	☐	☐	☐	☐	☐
...in terms of interpersonal aspects?	☐	☐	☐	☐	☐
...regarding discharge (medication, warning signs & recovery)?	☐	☐	☐	☐	☐
How satisfied were you with the availability of doctors for consultations?	☐	☐	☐	☐	☐

How satisfied were you with the accommodation, meals, and organization?

How satisfied were you with the organizational processes (scheduling and communication)?	☐	☐	☐	☐	☐
How satisfied were you with the room?	☐	☐	☐	☐	☐
How satisfied were you with the food (meals and drinks)?	☐	☐	☐	☐	☐
How satisfied were you with the planned length of stay?	☐	☐	☐	☐	☐
How satisfied were you with the staff-to-patient ratio?	☐	☐	☐	☐	☐

Final evaluation

How satisfied are you with the outcome of the surgery?	☐	☐	☐	☐	☐

Thank you very much for your cooperation. ☺
